# Guselkumab effectiveness in real-world settings: observations from an Italian multicentre study

**DOI:** 10.1093/rap/rkaf094

**Published:** 2025-09-08

**Authors:** Andrea Becciolini, Antonio Marchesoni, Simone Parisi, Alberto Lo Gullo, Olga Addimanda, Eleonora Celletti, Luca Idolazzi, Romina Andracco, Marino Paroli, Patrizia Del Medico, Antonella Farina, Palma Scolieri, Aurora Ianniello, Federica Lumetti, Cecilia Giampietro, Camilla Mazzanti, Alessandra Bezzi, Elisa Visalli, Elena Bravi, Alessandro Volpe, Rosetta Vitetta, Marta Priora, Viviana Ravagnani, Bernd Raffeiner, Aldo Biagio Molica Colella, Maddalena Larosa, Francesco Girelli, Veronica Franchina, Giulio Ferrero, Francesca Ometto, Valeria Nucera, Francesca Serale, Rosalba Caccavale, Mirco Magnani, Natalia Mansueto, Gianluca Smerilli, Maria Chiara Ditto, Riccardo Bixio, Maria Cristina Focherini, Fabio Mascella, Myriam Di Penta, Emanuela Sabatini, Alessia Fiorenza, Davide Murgia, Guido Rovera, Claudio Angrisani, Massimiliano De Simone, Giuditta Adorni, Eleonora Di Donato, Daniele Santilli, Roberta Foti, Ylenia Dal Bosco, Francesco De Lucia, Giorgio Amato, Francesco Molica Colella, Ilaria Platè, Vincenzo Bruzzese, Gerolamo Bianchi, Simone Bernardi, Antonio Marchetta, Rosario Foti, Gianluca Santoboni, Dario Camellino, Francesco Cipollone, Enrico Fusaro, Eugenio Arrigoni, Gianluca Lucchini, Gilda Sandri, Dilia Giuggioli, Massimo Reta, Alarico Ariani

**Affiliations:** Internal Medicine and Rheumatology Unit, University Hospital of Parma, Parma, Italy; Rheumatology Unit, Humanitas San Pio X, Milan, Italy; Rheumatology Department, Azienda Ospedaliera Universitaria Città della Salute e della Scienza di Torino, Turin, Italy; Rheumatology Unit, ARNAS Garibaldi di Catania, Catania, Italy; Rheumatology Unit, Azienda Unità Sanitaria Locale di Bologna—Policlinico S.Orsola-Azienda Ospedaliera Universitaria-IRCCS di Bologna, Bologna, Italy; Rheumatology Unit, “Clinica Medica” Institute, Ospedale SS. Annunziata di Chieti, G.d’Annunzio University of Chieti, Chieti, Italy; Rheumatology Section—Department of Medicine, AOUI Verona, Verona, Italy; Ambulatori di Reumatologia asl1 Liguria, Imperia Hospital, Imperia, Italy; Department of Clinical, Anesthesiological and Cardiovascular Sciences, Sapienza University of Rome, Rome, Italy; Rheumatology Outpatient Clinic—Internal Medicine Unit, Civitanova Marche Hospital, Civitanova, Italy; Internal Medicine Unit, Rheumatology Outpatient Clinic, Ospedale “A. Murri” di Fermo, Fermo, Italy; Department of Medical Specialties, “Nuovo Regina Margherita” Hospital, Rome, Italy; Rheumatology Outpatient Unit, ASL Novara, Novara, Italy; Rheumatology Unit, Azienda USL of Modena and University Hospital “Policlinico di Modena”, Modena, Italy; Rheumatology Outpatient Clinic, Azienda ULSS 6 Euganea, Padua, Italy; Center for the Diagnosis and Therapy of Autoimmune Rheumatological Diseases, Santa Rosa Hospital, ASL Viterbo, Viterbo, Italy; Internal Medicine and Rheumatology Unit, ASL Romagna, Rimini, Italy; Rheumatology Unit, Policlinico San Marco Hospital of Catania, Catania, Italy; Rheumatology Unit, Ospedale G. Da Saliceto, Piacenza, Italy; Rheumatology Unit, IRCCS Sacro Cuore Don Calabria Hospital, Negrar di Valpolicella, Verona, Italy; Unit of Rheumatology, Sant’Andrea Hospital, Vercelli, Italy; Rheumatology Unit, Rheumatology Day Hospital and Outpatient Clinic, Cuneo, Italy; Rheumatology Unit, Santa Chiara Hospital APSS, Trento, Italy; Department of Rheumatology, Teaching Hospital of the Paracelsius Medical University, Central Hospital of Bolzano (ASAA-SABES), Bolzano, Italy; Rheumatology Unit, Azienda Ospedaliera Papardo, Messina, Italy; Division of Rheumatology—Medical Specialties Department, Ospedale La Colletta-Azienda Sanitaria Locale 3, Genoa, Italy; Rheumatology Unit, Ospedale GB Morgagni—L Pierantoni, Forlì, Italy; Medical Direction, Papardo Hospital, Messina, Italy; Unit of Diagnostic and Interventional Radiology, Santa Corona Hospital, Pietra Ligure, Italy; Rheumatology Outpatient Clinic, Azienda ULSS 6 Euganea, Padua, Italy; Rheumatology Outpatient Unit, ASL Novara, Novara, Italy; Rheumatology Unit, Rheumatology Day Hospital and Outpatient Clinic, Cuneo, Italy; Department of Clinical, Anesthesiological and Cardiovascular Sciences, Sapienza University of Rome, Rome, Italy; Rheumatology Unit, Azienda Unità Sanitaria Locale di Bologna—Policlinico S.Orsola-Azienda Ospedaliera Universitaria-IRCCS di Bologna, Bologna, Italy; Ambulatori di Reumatologia asl1 Liguria, Imperia Hospital, Imperia, Italy; Rheumatology Outpatient Clinic—Internal Medicine Unit, Civitanova Marche Hospital, Civitanova, Italy; Rheumatology Department, Azienda Ospedaliera Universitaria Città della Salute e della Scienza di Torino, Turin, Italy; Rheumatology Unit, IRCCS Sacro Cuore Don Calabria Hospital, Negrar di Valpolicella, Verona, Italy; Internal Medicine and Rheumatology Unit, ASL Romagna, Rimini, Italy; Internal Medicine and Rheumatology Unit, ASL Romagna, Rimini, Italy; Rheumatology Unit, “Clinica Medica” Institute, Ospedale SS. Annunziata di Chieti, G.d’Annunzio University of Chieti, Chieti, Italy; Rheumatology Unit, “Clinica Medica” Institute, Ospedale SS. Annunziata di Chieti, G.d’Annunzio University of Chieti, Chieti, Italy; Unit of Rheumatology, Sant’Andrea Hospital, Vercelli, Italy; Unit of Rheumatology, Sant’Andrea Hospital, Vercelli, Italy; Unit of Rheumatology, Sant’Andrea Hospital, Vercelli, Italy; Center for the Diagnosis and Therapy of Autoimmune Rheumatological Diseases, Santa Rosa Hospital, ASL Viterbo, Viterbo, Italy; Center for the Diagnosis and Therapy of Autoimmune Rheumatological Diseases, Santa Rosa Hospital, ASL Viterbo, Viterbo, Italy; Internal Medicine and Rheumatology Unit, University Hospital of Parma, Parma, Italy; Internal Medicine and Rheumatology Unit, University Hospital of Parma, Parma, Italy; Internal Medicine and Rheumatology Unit, University Hospital of Parma, Parma, Italy; Rheumatology Unit, Policlinico San Marco Hospital of Catania, Catania, Italy; Rheumatology Unit, Policlinico San Marco Hospital of Catania, Catania, Italy; Rheumatology Unit, Policlinico San Marco Hospital of Catania, Catania, Italy; Rheumatology Unit, Policlinico San Marco Hospital of Catania, Catania, Italy; Internal Medicine Unit, Università Bicocca, Milan, Italy; Rheumatology Unit, Ospedale G. Da Saliceto, Piacenza, Italy; Department of Medical Specialties, “Nuovo Regina Margherita” Hospital, Rome, Italy; Division of Rheumatology—Medical Specialties Department, Ospedale La Colletta-Azienda Sanitaria Locale 3, Genoa, Italy; Rheumatology Unit, Ospedale GB Morgagni—L Pierantoni, Forlì, Italy; Rheumatology Unit, IRCCS Sacro Cuore Don Calabria Hospital, Negrar di Valpolicella, Verona, Italy; Rheumatology Unit, Policlinico San Marco Hospital of Catania, Catania, Italy; Center for the Diagnosis and Therapy of Autoimmune Rheumatological Diseases, Santa Rosa Hospital, ASL Viterbo, Viterbo, Italy; Division of Rheumatology—Medical Specialties Department, Ospedale La Colletta-Azienda Sanitaria Locale 3, Genoa, Italy; Rheumatology Unit, “Clinica Medica” Institute, Ospedale SS. Annunziata di Chieti, G.d’Annunzio University of Chieti, Chieti, Italy; Rheumatology Department, Azienda Ospedaliera Universitaria Città della Salute e della Scienza di Torino, Turin, Italy; Rheumatology Unit, Ospedale G. Da Saliceto, Piacenza, Italy; Internal Medicine and Rheumatology Unit, University Hospital of Parma, Parma, Italy; Rheumatology Unit, University of Modena and Reggio Emilia, Modena and Reggio Emilia, Italy; Rheumatology Unit, University of Modena and Reggio Emilia, Modena and Reggio Emilia, Italy; Rheumatology Unit, Azienda Unità Sanitaria Locale di Bologna—Policlinico S.Orsola-Azienda Ospedaliera Universitaria-IRCCS di Bologna, Bologna, Italy; Internal Medicine and Rheumatology Unit, University Hospital of Parma, Parma, Italy

**Keywords:** psoriatic arthritis, guselkumab, drug retention rate, real-world effectiveness

## Abstract

**Objectives:**

Guselkumab is a biologic disease-modifying antirheumatic drug (bDMARD) with proven efficacy for psoriatic arthritis (PsA) in randomized controlled trials. Evidence of its effectiveness from clinical practice remains limited. We evaluated the real-world effectiveness of guselkumab for PsA (primary objective) and identified factors influencing clinical outcomes.

**Methods:**

This retrospective, observational, multicentre study enrolled consecutive patients with PsA prescribed guselkumab for joint involvement at 26 Italian rheumatology referral centres. Baseline data included patient history, PsA subtype, treatment history and disease activity. Treatment effectiveness was assessed with Kaplan–Meier curves; Cox proportional hazards analysis identified factors associated with treatment persistence.

**Results:**

The study included 278 patients (median age: 57 years [interquartile range, IQR: 50–63]; 64.4% female); median observation 10.7 months (IQR: 5.3–15.9; total: 3332.6 patient-months). Retention rates at 6, 12 and 24 months were 90.4%, 80.0% and 67.8%, respectively. Reasons for discontinuation included primary inefficacy (48% of 54 cases), secondary inefficacy (41%) and skin/mucosal intolerance (4%). Statistically significant factors (*P *< 0.05) influencing treatment persistence included sex, smoking, concurrent conventional synthetic DMARDs (csDMARDs), corticosteroid use, year of prescription and axial or enthesitic involvement.

**Conclusions:**

Approximately two-thirds of PsA patients treated with guselkumab remained on therapy after 2 years. Adverse events motivated <10% of discontinuations. Effectiveness was higher in patients with enthesitic or axial PsA and in those without concurrent corticosteroids or csDMARDs, confirming the effectiveness and safety of guselkumab as an optimal choice for monotherapy, particularly in PsA patients with enthesitis, with or without joint impairment, and/or axial involvement.

Key messages

- Guselkumab was efficacious for PsA in controlled trials, but evidence of real-world effectiveness remains limited.
- We evaluated the real-world effectiveness of guselkumab for PsA and identified factors influencing clinical outcomes.
- Two-year treatment retention was approximately two-thirds, with adverse events motivating <10% of discontinuations.

## Introduction

Guselkumab is a biologic disease-modifying antirheumatic drug (bDMARD) that inhibits interleukin-23 (IL-23) signalling by selectively targeting the p19 subunit, blocking its interaction with the IL-23 receptor. It has been available to rheumatologists for the treatment of psoriatic arthritis (PsA) since December 2021 [1], and its use has progressively spread in this clinical context. Registrational clinical trials have demonstrated significant efficacy in most of the domains, such as joint and enthesitis, across a wide range of patients [2–4]. Approximately half of the patients with articular involvement in the DISCOVER 1 and 2 trials achieved low disease activity at 100 weeks of treatment [5], while resolution of enthesitis was observed in nearly 58% of enrolled subjects at 52 weeks [6].

However, the generalizability of these findings to real-world settings is limited by relatively short observation periods for a chronic condition, adherence to rigid treatment protocols, reliance on surrogate endpoints and the selected study populations that may not represent the broader clinical population [7, 8].

Initial real-world evidence on patients with PsA was primarily derived from studies on PsA patients in psoriasis (PsO) registries or real-life observational studies [9–11]. While these studies provide valuable insights into specific patient subsets, they lack the breadth to draw conclusions applicable to the broader PsA population encountered in routine clinical practice. Furthermore, existing real-world studies focusing on PsA populations are often limited by either short follow-up durations [12–15] or small sample sizes [16, 17]. Thus, there remains a critical need for studies that evaluate the medium- to long-term (≥2 years) performance of guselkumab in large cohorts of PsA patients treated in routine clinical practice.

This study assessed the effectiveness of guselkumab through its retention rate in patients with PsA treated in a real-world setting. Secondary objectives included recording the reasons for discontinuation and identifying predictors of treatment persistence.

## Methods

### Study design

This retrospective observational study assessed the 2-year retention rate of guselkumab. The study was conducted in accordance with the Declaration of Helsinki and approved by the Area Vasta Emilia Nord (AVEN) Ethics Committee, protocol code 34713.

### Patients

Participating centres included both hospital and university rheumatology units, representing a diverse range of healthcare facilities, including local centres. This heterogeneity was incorporated to ensure a comprehensive representation of PsA management in real-world clinical practice across Italy. Consecutive patients with PsA from 26 Italian centres were screened between October 2019 and December 2024. The inclusion criteria were: (a) diagnosis of PsA according to CASPAR criteria [18], (b) prior exposure to guselkumab and (c) availability of complete data. Patients receiving guselkumab exclusively for dermatological indications were excluded.

### Data collection

Demographic and clinical characteristics were recorded for each patient, including age, sex, smoking status, BMI, HLA-B27 positivity and disease duration. PsA domains involved were categorized as oligoarthritis, polyarthritis, axial disease, enthesitis and/or dactylitis. PSO severity: PsO extent was classified based on body surface area as 0%, <10%, 10–20% or >20%. Comorbidities of interest included inflammatory bowel disease, fibromyalgia and other conditions relevant for calculating the modified Rheumatic Disease Comorbidity Index (mRDCI) [19].

Data on treatment history included guselkumab start and discontinuation dates, as well as any use in the preceding 6 months of conventional synthetic DMARD (csDMARDs), bDMARDs, oral corticosteroids and/or intra-articular corticosteroid injections.

Assessment of disease activity at baseline and follow-up visits included tender/swollen joint count, number of painful entheses on the Leeds Enthesitis Index (LEI), Disease Activity Index for Psoriatic Arthritis (DAPSA), patient global assessment, dactylitis count [20], as well as the Bath Ankylosing Spondylitis Disease Activity Index (BASDAI) [21], C-reactive protein levels and a 10-point visual analogue scale for pain, as appropriate.

The reasons for treatment discontinuation were categorized as lack of efficacy, loss of efficacy, infections, malignancies or adverse cutaneous/mucosal reactions. Discontinuations because of remission, pregnancy or dermatological issues related to worsening of PsO were censored.

### Statistical analysis

The non-parametric distribution of data was assessed using the Pearson-D’Agostino normality test. Data are presented as median and interquartile range (IQR) or percentages, as appropriate.

The Kaplan–Meier method was used to graphically represent therapy retention rates. Cox proportional hazards analysis was performed to compute guselkumab retention rates and to identify factors associated with treatment persistence, including sex, age, BMI, concomitant csDMARD or corticosteroid use, PsA subtype, smoking status, treatment line and year of guselkumab prescription. A *P-value* <0.05 was considered statistically significant.

## Results

A total of 278 patients were enrolled in the study, with a median observation period of 10.7 months [IQR 5.3–15.9], corresponding to 3332.6 patient-months of follow-up. Baseline patient characteristics are summarized in Table 1. Overall, approximately one-third of patients had previously used at least two csDMARDs, and nearly half (49.6%) initiated guselkumab after failing at least two bDMARDs. Only 21.2% of patients received guselkumab as first-line therapy.

**Table 1.** Patient characteristics at baseline (*N* = 278)

Characteristic		
**Male prevalence, %**		35.6
**Age, median (IQR), years**		57 (50–63)
**Smokers, %**	Current	18.7
	Former	10.4
	Never	69.1
	Unknown	1.8
**BMI, median (IQR), kg/m^2^** ^a^		26.0 (23.2–29.4)
**PsA duration, median (IQR), months**		75 (38–126)
**mRDCI, median (IQR)** ^a^		1 (0–3)
**Inflammatory bowel disease prevalence, %**		4.3
**Fibromyalgia prevalence, %**		20.1
**Human leukocyte antigen B27, %**	Yes	3.6
	No	55.0
	Unknown	41.4
**MRI sacroiliitis, %**	Yes	28.8
	No	60.1
	Unknown	11.2
**Swollen joint count, median (IQR)**		2 (1–5)
**Tender joint count, median (IQR)**		7 (4–10)
**LEI, median (IQR)**		0 (0–2)
**Dactylitis severity score, median (IQR)**		0 (0–0)
**C-reactive protein, median (IQR), mg/dl**		1.0 (0.4–3.0)
**10-point visual analogue scale pain, median (IQR)**		8 (6–8)
**Patient global assessment, median (IQR)**		7 (5–8)
**DAPSA, median (IQR)**		26.3 (19.3–31.4)
**BASDAI, median (IQR)** ^b^		6.7 (5.5–7.6)
**PSO body surface area involvement, %**	0	32.0
	<10%	36.0
	10–20%	17.3
	>20%	13.7
	unknown	1.1
**Subset prevalence, %**	Oligoarticular	25.5
	Polyarticular	72.3
	Dactylitis	21.9
	Enthesitis	46.4
	Axial	35.3
**Line of treatment, median (IQR)**		2 (2–4)
**Prior conventional synthetic DMARD use, %**	Methotrexate	75.5
	Sulfasalazine	19.1
	Leflunomide	17.6
	Cyclosporin	16.2
**Prior biological DMARD use, %**	TNF inhibitor	68.7
	IL17 inhibitor	42.1
	IL12/23 inhibitor	11.2
	IL23 inhibitor	1.4
	CD80 inhibitor	1.8
**Prior targeted synthetic DMARD use, %**		9.5
**Prior locoregional treatment, %**		13.0
**Concomitant conventional synthetic DMARD, %**	Methotrexate	25.9
	Sulfasalazine	1.8
	Leflunomide	3.6
	Cyclosporin	0.4
	None	68.3
**Concomitant corticosteroids, %**		21.6

DAPSA, Disease Activity Index for Psoriatic Arthritis; BASDAI, Bath Ankylosing Spondylitis Disease Activity Index; LEI, Leeds Enthesitis Index; mRDCI, modified rheumatic disease comorbidity index; SJC, swollen joint count; PsA, psoriatic arthritis; IQR, interquartile range; TJC, tender joint count.

Data were missing in 19 (^a^) and 25 (^b^) patients.

**Figure 1.**
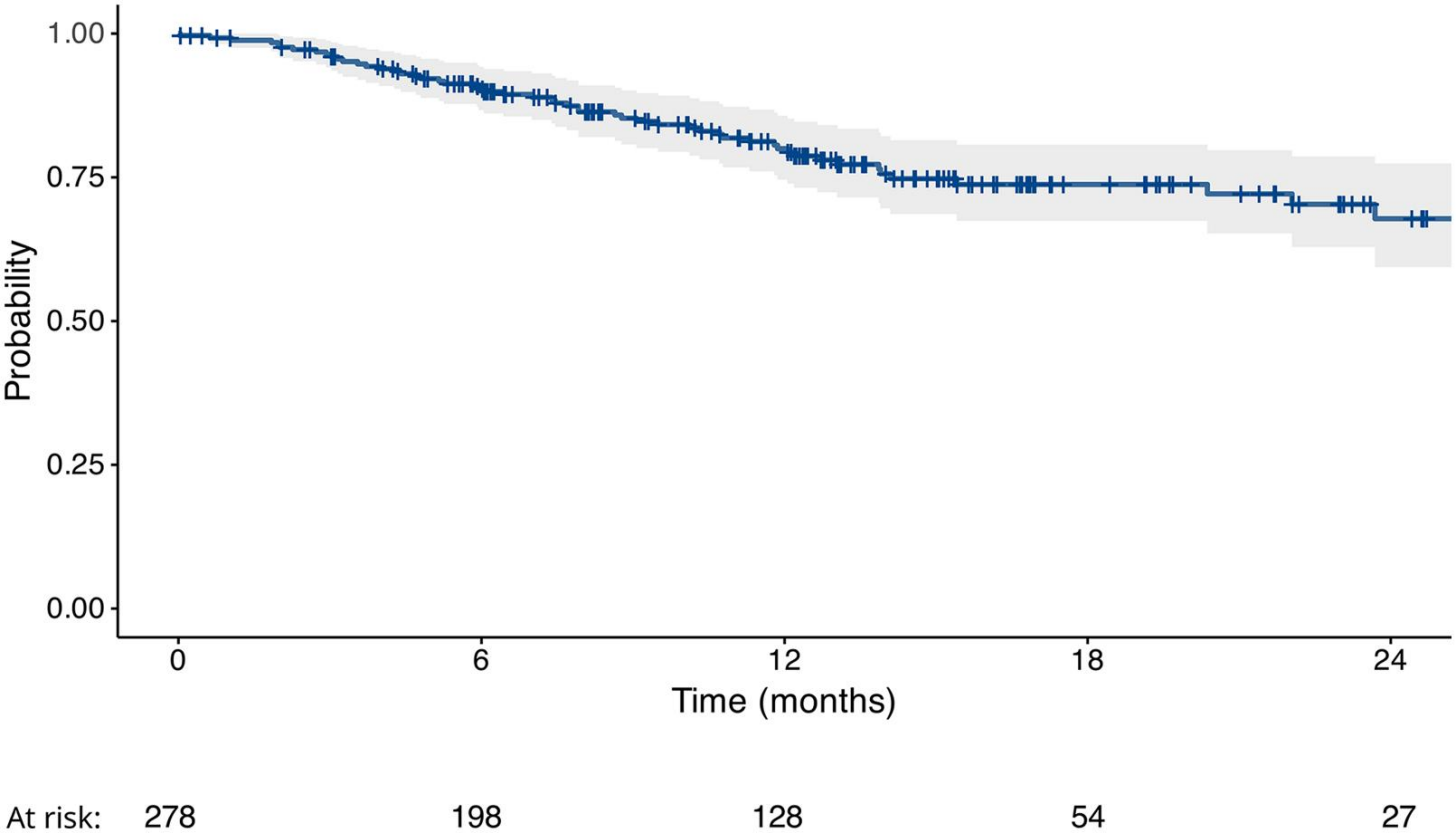
Kaplan–Meier estimates of the guselkumab retention rate over 24 months

The guselkumab retention rates at 6, 12 and 24 months were 90.4%, 80.0% and 67.8%, respectively (Fig. 1). Reasons for treatment discontinuation included primary inefficacy (48% of interruptions), secondary inefficacy (41%) and skin/mucosal intolerance (4%). Other reasons included infections, palpitations and cancer onset, each reported in one patient.

**Figure 2.**
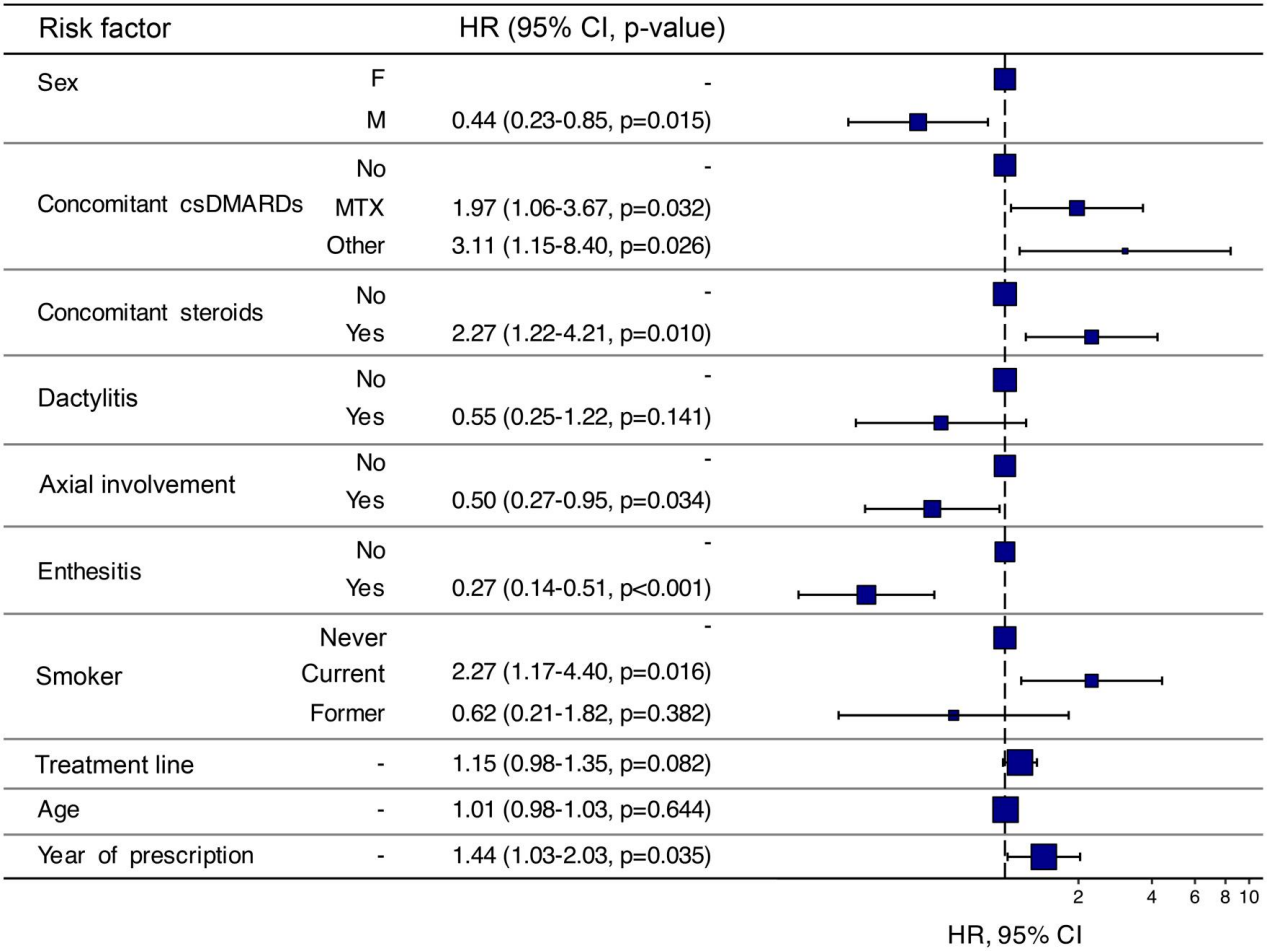
Cox proportional hazards analysis of covariate factors associated with guselkumab treatment persistence over 24 months

Multivariate analysis was initially performed considering all risk factors with minimal missing data, including age, concomitant csDMARD or corticosteroid use, PsA subtype, smoking status, treatment line and year of guselkumab prescription. The factors significantly influencing retention were sex, smoking status, year of prescription,

Factors significantly associated with longer guselkumab retention included male sex, and axial or entheseal involvement, while current smoking, more recent guselkumab prescription, concomitant csDMARD use and concomitant corticosteroid use were associated with shorter retention (Fig. 2).

In the subgroup of patients with available BMI data (*n* = 259), the same significant factors were identified except for year of prescription (data not shown). After 6 months of treatment, most patients maintained their baseline csDMARD (78.4%) and corticosteroid (79.5%) regimens.

## Discussion

To the best of our knowledge, this observational study is one of the largest and longest to assess the retention rate of guselkumab in a real-world PsA cohort. Accumulating evidence indicates that guselkumab is associated with a high retention rate, with pooled data from two guselkumab clinical trials showing that over 80% of patients complete at least 1 year of treatment per protocol [22].

The low frequency of severe adverse events observed in our cohort is consistent with pooled safety data on guselkumab from more than 4000 patients (>10 000 patient/years) in 11 phase II/III studies [23]; however, the excellent safety profile and low rate of adverse events [23] may only partially explain this high retention rate. Guselkumab is emerging as a drug that is associated with long-term improvement of multiple PsA domains [17, 24, 25]. The high retention rate may be considered an indicator of its effectiveness, more than its safety and tolerability.

Observational studies in patients with PsO have demonstrated that guselkumab exhibits a higher retention rate compared with other biologics, such as IL-17 inhibitors and TNF inhibitors [26]. Notably, the presence of arthritis does not appear to diminish treatment response in patients with primarily cutaneous disease [27]. Data from the BADBIR registry revealed that more than 90% of patients with PsA receiving guselkumab for PsO remained on treatment for 2 years [28]. However, among patients with both active PsA and PsO, the retention rate modestly decreases to just below 85% [10]. In patients with severe PsA who were diagnosed early and monitored within a dermatological cohort, the 1-year retention rate exceeded two-thirds [16]. These findings highlight differences in patient populations treated by dermatologists and rheumatologists, as the former typically encounter patients with predominant skin involvement, whereas rheumatologists more frequently manage cases where articular manifestations predominate, and skin involvement is less severe.

Previous studies primarily focused on PsO patients with concomitant arthritis, while investigations targeting PsA populations treated with guselkumab for joint disease are more recent and limited in sample size or follow-up duration. Data from the CorEvitas registry [15] showed high short-term (6-month) retention of ∼80%. Longer-term data from U.S. insurance registries indicate that guselkumab retention rates at 1 year surpass those of TNF inhibitors (∼70% vs 45%) [14] and IL-17 inhibitors (67% vs 50%) [15]. However, it is challenging to extrapolate these observations to different healthcare settings, as the reasons for treatment discontinuation are not always clear.

A multicentre study of a small, real-world PsA cohort managed directly by rheumatologists demonstrated a 12-month retention rate of ∼70% [17]; however, the long-term retention rate and factors influencing it remain poorly defined. Our study aimed to address these unmet needs.

The investigated cohort showed no significant differences in baseline characteristics such as BMI, disease duration, age, PsA subsets or disease activity compared with previously described cohorts [14, 15], except for the fact that nearly half of the patients had been treated with at least two prior bDMARDs, suggesting that this was a *difficult-to-treat* PsA population [29]. Guselkumab retention in our cohort after 1 year exceeded rates reported in clinical trials and aligned with other observational studies. At 2 years, there was a reduction in the incidence of treatment discontinuation (80% retained at 1 year vs just under 69% at 2 years).

The main factors influencing retention were consistent with the literature for other agents [30]. These included sex, smoking and year of guselkumab prescription. Concomitant corticosteroid or csDMARD use was associated with lower retention, while entheseal and axial involvement positively influenced retention. Corticosteroids are generally discouraged by EULAR guidelines [31] due to their potential rebound effects on PsO and PsA. The role of csDMARDs remains unclear [31], but our findings suggest that any concomitant csDMARD use (e.g. methotrexate, sulfasalazine, leflunomide or ciclosporin) might reduce guselkumab effectiveness.

Although this is the first report of such an observation for IL-23 inhibitors, a similar effect has been noted for other bDMARDs. For example, the PsABio study reported that methotrexate co-administration with ustekinumab (an IL-12/23 inhibitor) reduced its retention rate [31]. This may reflect a bias, as patients requiring combination therapy are often deemed at high risk of failure due to unmeasured clinical factors. Furthermore, variations in concomitant therapy during follow-up (e.g. NSAID use) were not systematically recorded. At the 6-month follow-up, however, 80% of patients had not altered their baseline concomitant treatment [31].

Guselkumab’s good performance in patients with entheseal involvement aligns with its mechanism of action, as IL-23 is highly expressed in entheses [32], and is supported by experimental studies [6]. Surprisingly, patients with axial PsA responded favourably to guselkumab. The axial involvement in these patients could have a different subtype of inflammation (i.e. enthesitis affecting the spine and pelvis), in contrast to patients with axial spondylarthritis who do not have concomitant PsO. For this reason, a novel score for the evaluation of axial involvement in PsA could be useful for to optimize disease management, as reported elsewhere [17]. Finally, we note that prior use of bDMARDs did not influence guselkumab effectiveness.

In addition to the limitations inherent to observational research, interpretation of the results of this study must consider several caveats. It was not possible to collect uniform detailed information on corticosteroids and NSAID dosages and changes over time; however, medical and legal restrictions likely limited long-term use of these agents. Disease subsets were classified by different evaluators across centres. Enthesitis was assessed using clinical or ultrasound criteria, while axial forms were frequently confirmed by MRI of the sacroiliac joints (in ∼90% of cases), suggesting that objective methods were commonly used. The lack of uniform disease activity measures across patients (e.g. LEI vs MASES index for enthesitis) precluded a unified analysis of disease activity as a retention factor. Finally, 7% of patients lacked BMI or mRDCI data, necessitating Cox regression analyses with and without BMI, which revealed minimal differences in the results.

In conclusion, this real-world study demonstrates the sustained effectiveness of guselkumab over time, even in patients previously treated with two bDMARDs. Key factors influencing retention included corticosteroid and/or csDMARD use (disadvantageous) and entheseal and/or axial involvement (advantageous). Considering its favourable safety profile, comparable to that observed in clinical trials, guselkumab is a viable treatment option for a broad range of PsA patients.

## Data Availability

Data available on request.

## References

[1] European Medicines Agency. Tremfya—summary of product characteristics. Turin: European Medicines Agency, n.d. https://www.ema.europa.eu/en/documents/product-information/tremfya-epar-product-information_en.pdf (3 January 2025, date last accessed).

[2] Deodhar A , Helliwell PS, Boehncke W-H et al; DISCOVER-1 Study Group. Guselkumab in patients with active psoriatic arthritis who were biologic-naive or had previously received TNFα inhibitor treatment (DISCOVER-1): a double-blind, randomised, placebo-controlled phase 3 trial. Lancet 2020;395:1115–25. 10.1016/S0140-6736(20)30265-8. 32178765

[3] Mease PJ , Rahman P, Gottlieb AB et al; DISCOVER-2 Study Group. Guselkumab in biologic-naive patients with active psoriatic arthritis (DISCOVER-2): a double-blind, randomised, placebo-controlled phase 3 trial. Lancet 2020;395:1126–36. 10.1016/S0140-6736(20)30263-4. 32178766

[4] Coates LC , Gossec L, Theander E et al Efficacy and safety of guselkumab in patients with active psoriatic arthritis who are inadequate responders to tumour necrosis factor inhibitors: results through one year of a phase IIIb, randomised, controlled study (COSMOS). Ann Rheum Dis 2022;81:359–69. 10.1136/annrheumdis-2021-220991. 34819273 PMC8862038

[5] Ritchlin CT , Mease PJ, Boehncke W-H et al Durable control of psoriatic arthritis with guselkumab across domains and patient characteristics: post hoc analysis of a phase 3 study. Clin Rheumatol 2024;43:2551–63. 10.1007/s10067-024-06991-8. 38844682 PMC11269379

[6] McGonagle D , McInnes IB, Deodhar A et al Resolution of enthesitis by guselkumab and relationships to disease burden: 1-year results of two phase 3 psoriatic arthritis studies. Rheumatology (Oxford) 2021;60:5337–50. 10.1093/rheumatology/keab285. 33822898 PMC8566200

[7] Pincus T , Bergman MJ, Yazici Y. Limitations of clinical trials in chronic diseases: is the efficacy of methotrexate (MTX) underestimated in polyarticular psoriatic arthritis on the basis of limitations of clinical trials more than on limitations of MTX, as was seen in rheumatoid arthritis? Clin Exp Rheumatol 2015;33:S82–S93. 26472658

[8] Alle G , Lopez-Medina C, Siebert S et al Patient profiles in randomized controlled trials versus a real-world study in psoriatic arthritis: scoping review and metaanalysis. J Rheumatol 2025;52:138–44. 10.3899/jrheum.2024-0653. 39547694

[9] Fitzgerald T , Melsheimer R, Lafeuille M-H et al Switching and discontinuation patterns among patients stable on originator infliximab who switched to an infliximab biosimilar or remained on originator infliximab. Biologics 2021;15:1–15. 10.2147/BTT.S285610. 33442230 PMC7797299

[10] Rocamora Vicenç , Crespi Laura, Ferran Marta et al Guselkumab effectiveness and survival in patients with psoriasis and psoriatic arthritis: multicenter analysis in daily clinical practice by the Spanish Psoriasis Group. Dermatol Ther 2022;35:e15865. 10.1111/dth.15865. 36175141

[11] Torres T , Varela P, Mendes Bastos P et al Tildrakizumab for the treatment of moderate-to-severe psoriasis: a 52-week, real-world Portuguese multicentric study. Drugs Context 2024;13:2023–12–5. 10.7573/dic.2023-12-5. PMC10954292 38510314

[12] Glintborg B , Gudbjornsson B, Krogh NS et al Impact of different infliximab dose regimens on treatment response and drug survival in 462 patients with psoriatic arthritis: results from the nationwide registries DANBIO and ICEBIO. Rheumatology (Oxford) 2014;53:2100–9. 10.1093/rheumatology/keu252. 24939677

[13] Werner SG , Baraliakos X, Reckert S et al Treatment with upadacitinib in active psoriatic arthritis: efficacy and safety data of the first 192 patients from the UPJOINT study, a multicentre, observational study in clinical practice. Rheumatol Ther 2023;10:1503–18. 10.1007/s40744-023-00589-3. 37695506 PMC10654267

[14] Walsh JA , Lin I, Zhao R et al Comparison of real-world on-label treatment persistence in patients with psoriatic arthritis receiving guselkumab versus subcutaneous tumor necrosis factor inhibitors. Drugs Real World Outcomes 2024;11:487–99. 10.1007/s40801-024-00428-z. 39083163 PMC11365907

[15] Mease PJ , Ogdie A, Tesser J et al Six-month persistence and multi-domain effectiveness of guselkumab in adults with psoriatic arthritis: real-world data from the Corevitas Psoriatic Arthritis/Spondyloarthritis Registry. Rheumatol Ther 2023;10:1479–501. 10.1007/s40744-023-00582-w. 37597159 PMC10654277

[16] Pantano I , Mauro D, Romano F et al Real-life efficacy of guselkumab in patients with early psoriatic arthritis. Rheumatology (Oxford) 2022;61:1217–21. 10.1093/rheumatology/keab509. 34152379

[17] Ruscitti Piero , Pantano Ilenia, Cataldi Giulia, et al Short-term effectiveness of guselkumab in psoriatic arthritis patients and suggestive features of axial involvement: results from a real-life multicentre cohort. Rheumatology (Oxford) 2025;64:1122–1130. 10.1093/rheumatology/keae220. 38598432

[18] Taylor W , Gladman D, Helliwell P et al; CASPAR Study Group. Classification criteria for psoriatic arthritis: development of new criteria from a large international study. Arthritis Rheum 2006;54:2665–73. 10.1002/art.21972. 16871531

[19] Spaetgens B , Wijnands JMA, van Durme C, Boonen A. Content and construct validity of the Rheumatic Diseases Comorbidity Index in patients with gout. Rheumatology (Oxford) 2015;54:1659–63. 10.1093/rheumatology/kev030. 25887028

[20] Mease PJ. Measures of psoriatic arthritis: Tender and Swollen Joint Assessment, Psoriasis Area and Severity Index (PASI), Nail Psoriasis Severity Index (NAPSI), Modified Nail Psoriasis Severity Index (mNAPSI), Mander/Newcastle Enthesitis Index (MEI), Leeds Enthesitis Index (LEI), Spondyloarthritis Research Consortium of Canada (SPARCC), Maastricht Ankylosing Spondylitis Enthesis Score (MASES), Leeds Dactylitis Index (LDI), Patient Global for Psoriatic Arthritis, Dermatology Life Quality Index (DLQI), Psoriatic Arthritis Quality of Life (PsAQOL), Functional Assessment of Chronic Illness Therapy-Fatigue (FACIT-F), Psoriatic Arthritis Response Criteria (PsARC), Psoriatic Arthritis Joint Activity Index (PsAJAI), Disease Activity in Psoriatic Arthritis (DAPSA), and Composite Psoriatic Disease Activity Index (CPDAI). Arthritis Care Res (Hoboken) 2011;63:S64–S85. 10.1002/acr.20577. 22588772

[21] Garrett S , Jenkinson T, Kennedy LG et al A new approach to defining disease status in ankylosing spondylitis: the Bath Ankylosing Spondylitis Disease Activity Index. J Rheumatol 1994;21:2286–91. 7699630

[22] Rahman P , Ritchlin CT, Helliwell PS et al Pooled safety results through 1 year of 2 phase III trials of guselkumab in patients with psoriatic arthritis. J Rheumatol 2021;48:1815–23. 10.3899/jrheum.201532. 33934076

[23] Strober B , Coates LC, Lebwohl MG et al Long-term safety of guselkumab in patients with psoriatic disease: an integrated analysis of eleven phase II/III clinical studies in psoriasis and psoriatic arthritis. Drug Saf 2024;47:39–57. 10.1007/s40264-023-01361-w. 37906417 PMC10764399

[24] McInnes IB , Rahman P, Gottlieb AB et al Long-term efficacy and safety of guselkumab, a monoclonal antibody specific to the p19 subunit of interleukin-23, through two years: results from a phase III, randomized, double-blind, placebo-controlled study conducted in biologic-naive patients with active psoriatic arthritis. Arthritis Rheumatol 2022;74:475–85. 10.1002/art.42010. 34719872 PMC9305108

[25] Coates Laura C , Gossec Laure, Zimmermann Miriam, et al Guselkumab provides durable improvement across psoriatic arthritis disease domains: post hoc analysis of a phase 3, randomised, double-blind, placebo-controlled study. RMD Open 2024;10:e003977. 10.1136/rmdopen-2023-003977. 38531621 PMC10966800

[26] Fitzgerald T , Zhdanava M, Pilon D et al Long-term psoriasis control with guselkumab, adalimumab, secukinumab, or ixekizumab in the USA. Dermatol Ther (Heidelb) 2023;13:1053–68. 10.1007/s13555-023-00910-6. 36929120 PMC10060501

[27] Torres T , Puig L, Vender R et al Drug survival of interleukin (IL)‑17 and IL‑23 inhibitors for the treatment of psoriasis: a retrospective multi‑country, multicentric cohort study. Am J Clin Dermatol 2022;23:891–904. 10.1007/s40257-022-00722-y. 35976568

[28] Yiu ZZN , Becher G, Kirby B et al; BADBIR Study Group. Drug survival associated with effectiveness and safety of treatment with guselkumab, ixekizumab, secukinumab, ustekinumab, and adalimumab in patients with psoriasis. JAMA Dermatol 2022;158:1131–41. 10.1001/jamadermatol.2022.2909. 35791876 PMC9260644

[29] Singla S , Ribeiro A, Torgutalp M, Mease PJ, Proft F. Difficult-to-treat psoriatic arthritis (D2T PsA): a scoping literature review informing a GRAPPA research project. RMD Open 2024;10:e003809. 10.1136/rmdopen-2023-003809. 38191215 PMC10806599

[30] Ariani Alarico , Santilli Daniele, Mozzani Flavio, et al Cycling or swap biologics and small molecules in psoriatic arthritis: observations from a real-life single center cohort. Medicine (Baltimore) 2021;100:e25300. 10.1097/MD.0000000000025300. 33879661 PMC8078287

[31] Gossec L , Kerschbaumer A, Ferreira RJO et al EULAR recommendations for the management of psoriatic arthritis with pharmacological therapies: 2023 update. Ann Rheum Dis 2024;83:706–19. 10.1136/ard-2024-225531. 38499325 PMC11103320

[32] Su Q-Y , Zhou H-N, Xia G-M et al Efficacy and safety of risankizumab in patients with psoriatic arthritis: a systematic review and meta-analysis of randomized controlled trials. Rheumatol Ther 2024;11:227–37. 10.1007/s40744-024-00638-5. 38302785 PMC10920559

